# Drug Utilization Study in Medical Emergency Unit of a Tertiary Care Hospital in North India

**DOI:** 10.1155/2014/973578

**Published:** 2014-05-05

**Authors:** Sharonjeet Kaur, Sujit Rajagopalan, Navjot Kaur, Nusrat Shafiq, Ashish Bhalla, Promila Pandhi, Samir Malhotra

**Affiliations:** ^1^Department of Pharmacology, PGIMER, Chandigarh 160012, India; ^2^Department of Internal Medicine, PGIMER, Chandigarh 160012, India

## Abstract

*Objective*. To generate data on the drug utilization pattern and cost of drug treatment and to determine the rationality of prescriptions. *Methods*. A retrospective cross-sectional drug utilization study was conducted in the medical emergency unit of our hospital. Patient case records were reviewed to extract data on the pattern of drug use. Cost of drug treatment for the emergency visit was calculated by referring to the cost mentioned in Monthly Index of Medical Specialties and the rationality of prescriptions was evaluated using WHO core indicators of drug utilization. *Results*. 1100 case records were reviewed. Majority of patients received proton pump inhibitors followed by multivitamins. The median cost per prescription was 119.23$ (7.32$–7663.46$). Majority (49.9%) of drug cost was driven by antibiotics alone. An average of 4.9 drugs was prescribed per prescription. There were 14.89% encounters with antibiotics. 75.17% of the drugs were given as injectables and only 29.27% of the drugs were prescribed as generics. *Conclusion*. There is need to rationalize the drug therapy in terms of increasing prescribing of drugs by generic name and to avoid overuse of PPIs and multivitamins in emergency unit. Also the hospital pharmacy should be encouraged to procure more cost effective alternative antibiotics in future.

## 1. Introduction

The World Health Organization (WHO) defines drug utilization research as “*the marketing, distribution, prescription and use of drugs in a society, with special emphasis on the resulting medical, social, and economic consequences*” [1]. Thus, inherent in the definition, such studies provide logical background for determining the rationality of drug use as well as providing evidence based guidance for making policy decisions at various levels of healthcare. Drug utilization research studies conducted in the inpatient settings are effective tools that help in evaluating the drug prescribing trends, efficiency, and cost-effectiveness of hospital formularies. There is always a variation in drug utilization among different countries and even among health institutions within a country and sometimes within the same institute at different point of time probably because of changing disease trends over a period of time [2]. Conducting periodic studies of pattern of drug use in various hospital settings or patient populations is therefore essential to critically analyse the current hospital drug policies and to make recommendations based on various guidelines to improve upon the current drug usage pattern in the future, if needed. This is more importantly required in resource poor countries like ours so as to ensure that the scarce resources are utilized in the best possible manner. Though there have been various drug utilization studies conducted on specific populations and in varied settings in India [3, 4], only a few have been conducted in emergency settings [5–7]. Previous studies conducted by the authors in our hospital emergency department were primarily safety utilization studies [8, 9].

The emergency department represents an important platform for conducting drug utilization studies as patients present with a wide range of diseases in acute form and the drug use is quite extensive. Therefore, evaluating the drug prescribing behaviour and usage patterns in the emergency settings has the potential of determining the rationality of drug therapy being given in the particular region to a broader extent. Keeping this in view, we conducted a drug utilization study in our tertiary care hospital with the objective of studying pattern of drug use and cost of drug treatment and determining the rationality of prescriptions so as to identify priority areas that need to be targeted for further improvement in patient care.

## 2. Methods

We conducted a retrospective cross-sectional drug utilization study at the Medical Emergency Department of Postgraduate Institute of Medical Education and Research, Chandigarh, India, over a period of six months. Our institute is a premier tertiary care hospital which caters to needs of a large patient pool from both urban and rural areas of North India. Medical emergency unit has 44 beds and has a turnover of about 3000 patients per month. All adult patients presenting with any emergency medical need are attended by internal medicine specialists in the emergency department for initial management.

For studying the drug utilization pattern, following data were collected-(i) age, (ii) gender, (iii) average stay in the emergency department, (iv) diagnosis of the patient, (v) comorbid conditions. Detailed information on drugs used including name of the drug, dosage schedule (form, route, and frequency), and duration of treatment was recorded from the patient medical records.

For cost assessment, we considered only those drugs which were prescribed in the emergency department for the presenting acute condition. Cost of individual drugs was calculated according to the pricing of drugs given in Monthly Index of Medical Specialists (MIMS). We summed up the costs for each group of drugs. Cost of individual prescriptions was also calculated. The currency we used for cost calculations was INR, which was later converted to US dollars (2011).

Rationality of prescriptions was evaluated by using the WHO core drug prescribing indicators, that is, (a) average number of drugs per encounter, (b) percentage of encounters with an antibiotic, (c) percentage of encounters with an injection, (d) percentage of drugs prescribed from the essential drugs list or formulary, and (e) percentage of drugs prescribed by generic names. Indian National List of Essential Medicines, 2003, was used for assessing the number of drugs prescribed from the essential list. The term antibiotic was inclusive of antitubercular, antiprotozoal, and antihelminthic agents in addition to antibacterial, antiviral, and antifungal agents.

Statistical analysis—continuous data are expressed as mean ± S.D. Nominal data were expressed as percentages. No formal statistical hypothesis was tested.

## 3. Results

### 3.1. Analysis of Drug Utilization Pattern

A total of 1100 case records were reviewed. Among the cases, 736 were males and 364 were females. The mean age group of patients admitted was 46 ± 17.4 years. Among the different age groups of the patients admitted, maximum patients (*n* = 452) were in the age group of 40 to 59 yrs. The average stay of patients in the emergency department was 2.23 ± 1.3 days.

**Figure 1:**
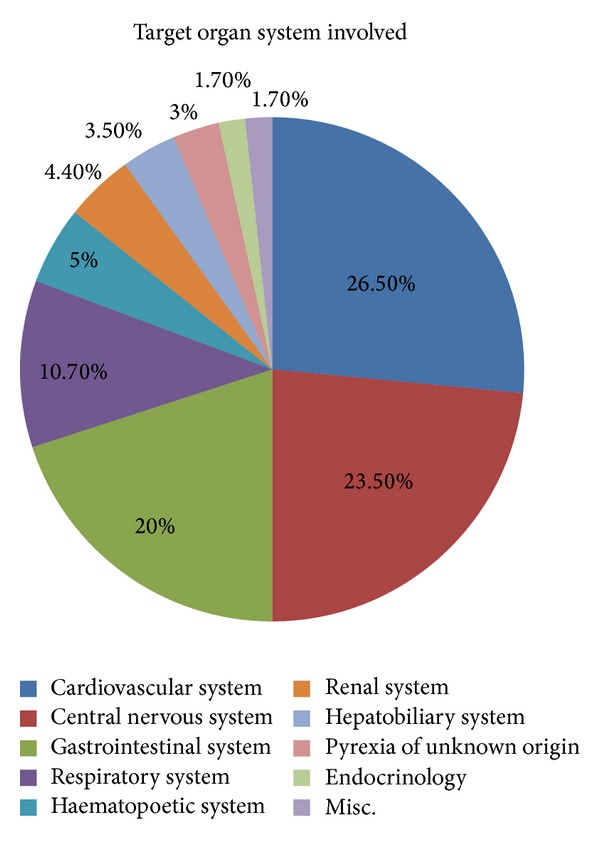
Pie chart showing the distribution of various target organ systems involved.

Majority of the patients presented with diseases of cardiovascular system (26.5%), followed by central nervous system (23.5%), gastrointestinal system (20%), and respiratory system (10.7%). The rest of the cases were related to other organ systems as given in Figure 1. Acute coronary syndrome (13.67%), seizures (7.14%), acute cerebrovascular accidents (5.45), acute gastroenteritis (5.09), and epigastric pain presenting as acid peptic disease (5%) followed by hypertensive disease and respiratory disorders like acute exacerbation of chronic obstructive pulmonary disease and asthma constituted the majority of the total cases. Hypertension and diabetes mellitus (49.69%) accounted for the majority of the underlying comorbid conditions, followed by chronic kidney disease, coronary artery disease, tuberculosis, COPD, liver disease, dilated cardiomyopathy, cancer, and arrhythmias (Table 1).

**Figure 2:**
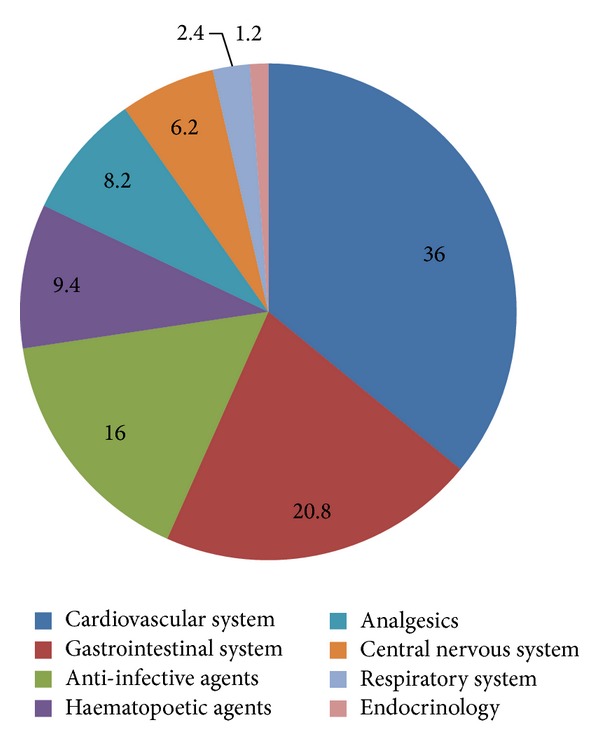
Pie chart showing various classes of drugs prescribed.

**Figure 3:**
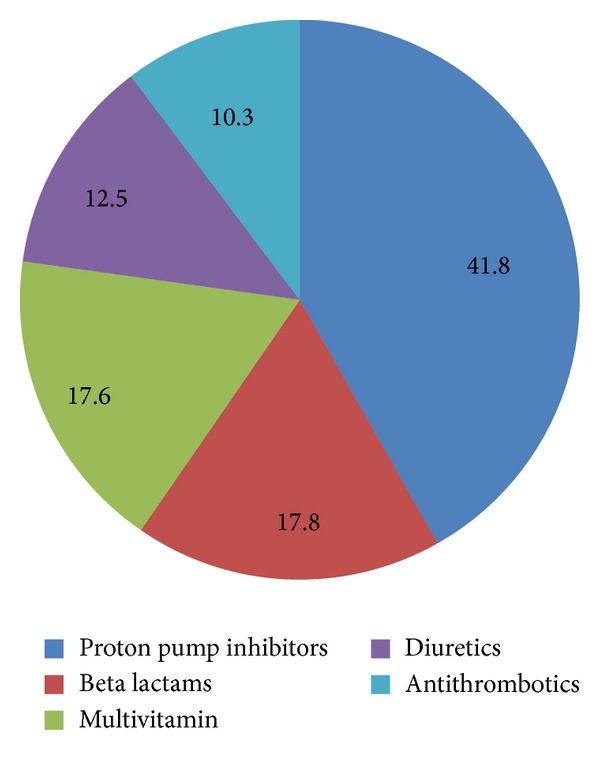
Percentage of the drugs used as injectables.

Most of the drugs prescribed were for cardiovascular system (36%) followed by gastrointestinal system (20.8%) and other systems (Figure 2). Among the drugs prescribed, majority of the patients (*N* = 646) received proton pump inhibitors, followed by multivitamins (*N* = 567), antibiotics (*N* = 462), diuretics (*N* = 265), and antiplatelet drugs (*N* = 224) (Table 2). 1.44% of the total prescribed drugs were given as fixed dose combinations (FDCs). The commonly prescribed FDCs were amoxicillin-clavulanic acid (0.83%), antitubercular therapy (0.20%), piperacillin-tazobactam (0.16%), and trimethoprim-sulphamethoxazole (0.08%). Among the injectable preparations proton pump inhibitors (41.8%) were the most commonly prescribed agents followed by other agents as shown in Figure 3.

### 3.2. Cost of Drug Treatment

Cost of individual drug classes varied widely. The summed-up costs for each group of drugs towards out-of-pocket expenses are presented in Table 3. Among the different drugs prescribed, anti-infectives (49.9%) accounted for the maximum cost followed by the drugs acting on the cardiovascular system (25.8%), drugs acting on the gastrointestinal system (11%), nutritional supplements (4.7%), analgesic/anti-inflammatory drugs (4.3%), and hormones (2.8%).

### 3.3. Analysis of Prescription Indicators

Analysis of prescriptions using WHO core indicators revealed that the average number of drugs prescribed per prescription was 4.9. There were 610 (14.89%) encounters with antibiotics. 75.17% of the drugs were prescribed as injectables. 64.94% of the total drugs were prescribed from the national essential medicine list, 2003. Branded drugs constituted 70.73% and generic drugs constituted 29.27% of the total drugs prescribed (Table 4).

## 4. Discussion

The emergency department of a tertiary care unit of a developing country is faced with the problem of heavy patient load and relative paucity of human and economic resources. Specifically, our hospital is a premier tertiary care hospital which caters to a large population pool of the North Indian region. The drugs are prescribed by the attending internal medicine physician. The emergency drugs are provided within the hospital but the majority of the drug expenses have to be borne by the patients themselves.

The average stay of the patients in the emergency was 2.23 ± 1.3 days. This indicates a rapid and efficient management of patients after which they were discharged or they were transferred to a medical ward. The most commonly involved organ systems were cardiovascular system and CNS and the top five diseases were acute coronary syndrome, seizures, acute cerebrovascular accidents, acute gastroenteritis, and acute epigastric pain. This picture is representative of our medical emergency set-up which caters to wide range of diseases presenting in acute forms. The most common underlying comorbid conditions were hypertension and diabetes mellitus which adds to the existing body of evidence that these diseases are assuming epidemic proportions in developing countries as well.

Analysis of case records for drug utilization pattern revealed that most of the drug classes were prescribed for appropriate indication. However, prescription of multivitamins appears largely irrational as they are not indicated for the management of emergency conditions. Moreover, a large number of these agents were prescribed as injectables and since these are expensive, they would have definitely increased costs associated with the emergency visit. Similarly, proton pump inhibitors were also found to be used for some patients where there was no appropriate indication, for example, in a patient of fever under evaluation, in a patient of right thigh cellulitis, and in a patient of bronchiectasis. The rationality of use of proton pump inhibitors and multivitamins is thus questionable.

We considered only the direct drug costs for those agents that were prescribed for the presenting emergency condition. Cost of chronic medications was not considered in the analysis nor was that of the other hospital charges. The median cost per prescription was 119.23$ (7.32$–7663.46$). This is very high, especially in our setting where most of the patients are from the lower socioeconomic strata and cannot afford such expensive therapy. Analysis of the data revealed that majority of the cost was driven by antimicrobials. This is probably because, in an emergency setting, there is a dire need to give broad spectrum antibiotics as empirical therapy, which is usually very expensive. But what is essential is to narrow down the therapy as soon as we have a sensitivity report for the infecting organism. Apart from the benefit of reduced cost, this will also help combat drug resistance due to inadvertent use of antibiotics. In our hospital set-up, the culture sensitivity report usually becomes available within 48–72 hrs. So, for most of the patients de-escalation did not happen in the emergency visit but was advised at the time of discharge from emergency unit where it was required.

Average number of drugs per prescription was 4.9 which is more than double the average number (i.e., 2) recommended by WHO [10]. However, this cannot be considered irrational polypharmacy practice as there is need of empirical therapy till definitive diagnosis becomes clear and secondly for management of acute life threatening conditions most of the patients would usually require more than two drugs. Different studies conducted in India have given varied results, but all of them point to higher incidence of multiple drug usage in emergency set-ups. For example, one reports this incidence to be 3.3, whereas another study has reported the use of drugs to be as high as 9.9 ± 2.5 drugs per prescription [6, 11]. Similarly number of encounters with injectables was on the higher end (75.17%), which again seems justifiable on account of need of immediate drug action. Drugs prescribed from the WHO essential medicine list comprised only 64.94% of drugs. This proportion should have been higher since this list of drugs is prepared with regard to public health relevance, evidence on efficacy and safety of the drugs, and comparative cost effectiveness. Also, another area of concern was the lower proportion of drugs prescribed as generics (only 29.27%). There are several benefits of prescribing drugs as generics such as increased patient compliance and lower cost of drug therapy [12, 13]. American Academy of Family Physicians recommends prescribing drugs in generic forms as a strategy to avoid high cost of drug therapy [14].

Our study has some limitations. We are not certain if our sample size of 1100 patients was truly representative of total population visiting emergency, but nevertheless our sample size is much larger as compared to previous studies conducted in India [7, 11]. Another limitation was that we have not analysed the data for the disorders managed differently (e.g., acute coronary syndrome with or without ST segment elevation). This would have given a clearer picture on the cost of drug treatment.

## 5. Conclusion

This study highlights the need for rationalising drug therapy in the emergency settings with regard to increasing adherence to national essential medicine list and increasing prescription of drugs by generic name. Also there is need to prevent inappropriate overuse of PPI and multivitamins where it is not indicated. Since the drug cost is mostly driven by prescription of broad spectrum antibiotics, therefore hospital pharmacy should be encouraged to procure more cost effective alternative antibiotics in future.

## Figures and Tables

**Table 1:** Various comorbid conditions in the cases studied.

S. number	Underlying disease	Number of cases (%)
1	Hypertension	86 (26.06)
2	Diabetes mellitus	78 (23.63)
3	Chronic kidney disease	29 (8.78)
4	Coronary artery disease	19 (5.75)
5	Tuberculosis	16 (4.84)
6	Chronic obstructive pulmonary disease	15 (4.54)
7	Chronic liver disease	13 (3.93)
8	Dilated cardiomyopathy	12 (3.63)
9	Cerebrovascular accidents	10 (3.03)
10	Cancer	6 (1.81)
11	Arrhythmias	1 (0.30)

**Table 2:** Most commonly prescribed drugs.

Drug class	Number of patients	ATC
Proton pump inhibitors	646	A02BC
Multivitamins	567	A11
Antibiotic	462	J01
Diuretics	265	C03
Antiplatelet drug	224	B01AC

ATC: Anatomical Therapeutic and Chemical Classification drug class coding (as per WHO).

**Table 3:** Cost of various drug classes.

Drug class	Cost of drugs (USD)
Sulphonamide	0.261
Cholinergic	0.53
ARBs	1.59
Anticholinergic	7.32
H2 blockers	7.33
Tetracyclines	9.49
Antifungal	12.5
Beta blockers	14.56
Bronchodilators	15.12
NSAIDS	16.14
Aminoglycosides	21.42
Opioids	52.93
ACE inhibitors	55.49
Sedative hypnotics	69.78
Antitubercular drugs	85.22
CCBs	85.42
Statins	92.33
Antiplatelet	95.55
Inotropic agents	97.84
Misc. GIT drugs	119.23
Antiemetics	167.82
Antiviral	180.48
Misc. CVS drugs	196.86
Nitrates	199.54
Antifibrinolytic	220.28
Antiepileptic	236.81
Antiarrhythmics	298.44
Antidiabetic	316.57
Antiprotozoal	375.99
Fluoroquinolones	382.14
Fibrinolytic drugs	492.91
Hormones	575.51
Corticosteroids	792.1
Multivitamins	956.68
Diuretics	1032.46
PPIs	1913.61
Antithrombotic	2312.24
Macrolides	3605.81
Beta lactams	7663.46

**Table 4:** Analysis of prescriptions using WHO core prescribing indicators.

WHO core indicators	*N* (%)
Average number of drugs per prescription	4.9
Encounter with antibiotics	610 (14.89)
Encounter with injectables	3079 (75.17)
Drug prescribed from essential medicine list	2660 (64.94)
Drug prescribed as generic	1199 (29.27)
